# Study on the Influence Mechanism of Environmental Management System Certification on Enterprise Green Innovation

**DOI:** 10.3390/ijerph191912379

**Published:** 2022-09-28

**Authors:** Weizhou Su, Gaowen Lei, Sidai Guo, Hongche Dan

**Affiliations:** School of Economics and Management, Southwest University of Science and Technology, Mianyang 621010, China

**Keywords:** environmental management system certification, green innovation, difference-in-differences, enterprise heterogeneity

## Abstract

Improving the green technology innovation capability of enterprises is an important way for industrial enterprises to improve product quality and production efficiency and reduce industrial pollution and energy consumption. Based on the Porter hypothesis, this paper took the data of listed companies of the heavy polluting industry in Chinese A-shares from 2011–2018 as a study sample, and a difference-in-differences (DID) model was constructed to explore the impact of environmental management system certification (EMSC) on enterprises’ green innovation. This paper also studied the differential impact between the EMSC and enterprises’ green innovation from the perspective of enterprise heterogeneity. It was found that the EMSC has a significant promotion effect on the enterprises’ green innovation; this promotion changes with the size and ownership of the enterprise and the lifecycle of the enterprise. Meanwhile, customer, shareholder, and creditor satisfaction all play a positive moderating role in the process of EMSC affecting green innovation, while the moderating role of supplier satisfaction is not significant. The findings of this paper have important implications for the understanding of the role of EMSC in promoting green innovation in enterprises.

## 1. Introduction

The 2020 BP World Energy Yearbook shows that China is still the world’s largest energy-consuming market and CO2-emitting country. How to protect the ecological environment while promoting economic development is a major issue facing the Chinese government. The China National 14th Five-Year Plan has made a major strategic decision to “strive to achieve carbon peaking by 2030 and carbon neutrality by 2060”, and green technology innovation is essential to achieve “carbon peaking” and “carbon neutrality”. The important supporting role of green technology innovation in achieving the goals of “peak carbon” and “carbon neutral” has become a hot topic of discussion at present. In October 2015, Xi Jinping, General Secretary of the CPC Central Committee, put forward the new development philosophy of “innovation, coordination, green, openness and sharing” at the Fifth Plenary Session of the 18th CPC Central Committee, placing innovation and green concepts in an important position. “Green Development” and the requirement to “build a market-oriented green technology innovation system” were clearly stated at the 19th Communist Party Congress of China in October 2017. In April 2019, to further implement the Party Central Committee’s deployment and top-level design of green technology innovation, the China National Development and Reform Commission and the Ministry of Science and Technology issued the “Guidance on Building a Market-Oriented Green Technology Innovation System”, the guidance further refining the roadmap and timetable for the construction of a green technology innovation system. In October 2020, “China’s 14th Five-Year Plan” proposed to accelerate “green and low-carbon development” and “support green technology innovation” as the development goal. It is evident that green technology innovation has become an important task in the construction of the national ecological civilization in China.

How to quickly enhance the ability of green technology innovation, promote industrial enterprises to improve product quality and production efficiency, reduce resource and energy consumption, reduce pollution emissions or even achieve zero emissions, in the current industrial development in China is particularly important. Existing research on green innovation has been conducted from two perspectives: regional and industry as well as firm level. At the regional level, scholars have mainly focused on how to promote regional green innovation by means of environmental regulation [1,2,3,4], although some scholars have also noticed other factors in the development of regional green innovation, for example [5], analyzed the influence of network development, entrepreneurship on regional green innovation [6], analyzed the potential influence of industrial agglomeration, marketization [7], analyzed the regional environmental performance, the potential impact of green finance,3 and others have also noted the potential impact of green finance. At the firm level, scholars have noted the influence of factors such as firms’ environmental sustainability strategies [8], loaning size, government subsidies, knowledge management, credit policies, and carbon emissions trading on firms’ green innovation [9,10,11,12], but fewer scholars have paid attention to how environmental management system certification (EMSC) affect firms’ green innovation.

ISO14001, an environmental management system certification (EMSC) standard promulgated by the International Organization for Standardization in 1996 on the basis of ISO9000 quality management system standard, was converted to a national standard in China in 1997 under the symbol GB/T24000-ISO14001. The national standard combines the economic and environmental objectives of an enterprise, this is an important guide to help enterprises establish a scientific environmental management system. Environmental management system certification (ISO14001) (EMSC), an important supplement to the traditional means of environmental governance, raises the awareness of environmental protection among all people, strengthens the concept of the environmental legal system, realizes the rational use of resources, and protects the environment for human survival and development. Meanwhile, the implementation of ISO14001 is also conducive to the transformation of enterprises from sloppy management of production methods to efficiency management, from post-event management of the environment to pre-event prevention and control, enhancing the public image of enterprises themselves and the rights and interests of shareholders [13], It can also help enterprises to strengthen their competitive edge, enter market segments and gain higher profits [14,15,16].

In order to explore the influence of EMSC on corporate green innovation and further investigate its specific influence mechanism, this paper token the data of listed companies of the heavy polluting industry in Chinese A-shares from 2011–2018 as a study sample, a difference-in-differences (DID) model was constructed to explore the causality between EMSC and enterprises’ green innovation. In addition, this paper also studied the differential impact of EMSC on enterprises’ green innovation based on the different enterprise sizes, nature of ownership, and life cycle. Furthermost, this paper also introduces two different types of stakeholder roles, self-focused and mutually beneficial, to examine the interaction effects of stakeholders and EMSC on corporate green innovation.

The rest of this paper is arranged as follows: Section 2 introduces literature review and the research hypotheses; Section 3 introduces the data and methodology; Section 4 presents the results of the empirical test; finally, the conclusions and policy recommendations are offered in Section 5.

## 2. Literature Review and Research Hypothesis

### 2.1. Literature Review

**Figure 1:**
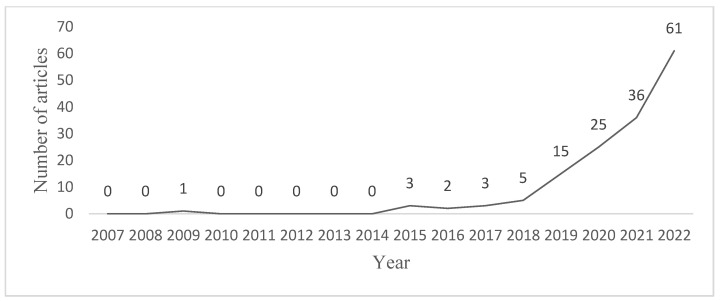
Keywords trends in environmental regulation.

In this section, a systematic literature review was conducted using bibliometric methods with CiteSpace software (version 6.1.R3, Chaomei Chen, Philadelphia, PA, USA), and the current status of research on corporate green innovation and its development direction was obtained using keyword co-occurrence. Specifically, search the Web of Science database on the topic of corporate green innovation, then 1117 papers were obtained from journals related to the topic during 2007–2022. The 1117 papers were imported into CiteSpace in plain text format, and the node type was selected as “keywords”, and the source of the subject words was selected as “title” and “abstract”. The top 20 keywords were listed, as shown in Table 1, and the highest frequency of performance was 310 times, and the year of its first appearance was 2008; the frequency of innovation and impact are 282 and 275 respectively. Moreover, green innovation, management, corporate social responsibility, environmental regulation green, and eco-innovation also have a high frequency of occurrence. Among these keywords, environmental regulation is similar to the research topic of this paper, so we further paid attention to the trends of the keyword environmental regulation and obtained the results shown in Figure 1. From Figure 1, we can see that among the studies related to green innovation of enterprises, environmental regulation has gradually attracted the interest of scholars since 2016 and has become a major research hotspot at present. After analyzing the relevant literature, it is found that the current status of research on environmental regulation and corporate green innovation is as follows.

In the literature related to the impact of environmental regulation on green innovation, Liao et al. (2018) found that environmental regulation, fiscal and tax measures are the main factors that affect green innovation in firms after analyzing the various influencing factors of green innovation in firms [17]. On the question of how environmental regulation affects green innovation, Wang et al. (2020) and Yi et al. (2020) both found that environmental regulation promotes firms’ green innovation [18,19], while Liu et al. (2021) also found that the implementation of environmental regulation policies leads firms to tend to apply for more green patents by analyzing data from Chinese listed companies [20], and Yang et al. (2021) found that direct environmental regulation in the region stimulates firms to engage in green innovation [21]. In addition, Zhong and Peng (2022) found that the implementation of environmental regulation policies significantly promoted green innovation among firms in heavily polluting industries, and the marginal effect of this positive effect showed a decline and then an increase over time [22]. In terms of the mechanism of action of environmental regulation to promote green innovation of enterprises, Sun et al. (2019) found that environmental regulation led to an increase in environmentally friendly R&D investment of enterprises, which in turn promoted green technological progress of enterprises [23], Peng et al. (2021) found that environmental regulation could promote green innovation behavior through structural equation modeling, and there was a moderating effect of green innovation intention in the process [24]. However, some scholars have also found different conclusions related to the impact of environmental regulation on the green innovation of enterprises. He et al. (2019) found there is no significant effect of environmental regulation on firms’ green innovation through structural equation modeling [25], Pan et al. (2020) found an inverted U-shaped relationship between environmental regulation and green innovation by analyzing data from Chinese regions [4], Tang et al. (2020) find that environmental regulations inhibit firms’ green innovation in the short run by reducing firms’ cash flow [26], Yang and Wang (2021) find heterogeneity in the effects of environmental regulations on firms’ green innovation in different regions [27]. Therefore, there is no consistent conclusion on the impact of environmental regulation on corporate green innovation in current studies. As one of the environmental regulation tools, the impact of EMSC on enterprises’ green innovation needs to be verified through theoretical and empirical analysis.

### 2.2. Research Hypothesis

#### 2.2.1. The Relationship between EMSC and Corporate Green Innovation

As a voluntary environmental regulation tool [28], the impact of EMSC on corporate green innovation can be analyzed from the perspective of Porter hypothesis. The Porter hypothesis is a theory on the relationship between environmental regulation and innovation, it has been tested by a number of studies since its introduction. Among these studies, some scholars have shown that the Porter hypothesis is invalid in practice. For example, Lanoie et al. (2011) argue that environmental regulation leads to higher production costs and damages the competitiveness of firms, which is detrimental to green innovation [29]. Smirnova et al. (2021) also show that environmental regulation leads to a decrease in the output of MSMEs rather than promoting their green innovation level [30]. However, other scholars argue that environmental regulation is a necessary condition for firms to engage in green innovation, and that despite the fact that environmental regulation causes increased costs for firms, firms can obtain compensation through successful green innovation, and thus environmental regulation can trigger green innovation in firms [31,32]. In practice, Dechezlepretre and Sato (2017) found that well-designed environmental regulation policies promote green innovation [33], Leeuwen and Mohnen (2017) also found that either existing or expected environmental regulations positively promote firms’ green innovation [34]. Wang et al. (2022) found that environmental regulations triggered firm innovation by analyzing the Chinese steel industry 1995–2017 data [31].

In this paper, EMSC, a voluntary environmental regulation tool [28], can help to improve the transformation of green innovation technologies [35]. In addition, as a means of communicating information to companies [36], EMSC helps companies to diffuse differentiated information, helping them to win the trust of stakeholders and to gain more resources and financial support to develop more environmentally friendly products [37]. From a government management perspective, when an enterprise is certified to an environmental management standard, it will receive a range of support from the government, including financial, material, and human resources [38]. This will compensate for the cost of green innovation activities and facilitate the development of green innovation activities [28]. As a result, companies with EMSC have an incentive to improve their green innovation capabilities. Based on the above analysis, the following hypotheses are proposed in this study.

**H1:**  *EMSC can promote green innovation in business.*

#### 2.2.2. The Differentiated Impact of EMSC on Corporate Green Innovation

According to factor endowment theory, different features of the enterprise can play different role in the process of environmental regulation affecting green innovation, therefore, this study analyses the differentiated impact of EMSC on enterprise’s green innovation in terms of enterprise’s ownership, enterprise’s size and enterprise’s life cycle.

Due to the dual externality characteristic of green innovation activities [39], private enterprises often face higher innovation risks and are less likely to obtain high returns through green innovation than state-owned enterprises (SOEs). Market failures often arise if private enterprises are given the task of green technology R&D. Governments can often address some of these market failures by regulating the decisions of SOEs. Compared to private enterprises, SOEs have a policy mandate [40] and with a social orientation that places equal emphasis on environmental governance and economic development, the pressure on SOEs to save energy and reduce emissions is much greater than that on private enterprises. In addition, SOEs have a greater advantage in the allocation of resources, which reduces the risk of their green innovation activities [41]. Therefore, when reasonable environmental regulations exist, SOEs are more willing to engage in green innovation activities than private enterprises.

The risk-taking capacity of enterprises is an important factor influencing their green innovation activities [42]. Green innovation activities require significant R&D costs, and the return on investment is long and risky, large enterprises have a greater risk-taking capacity than small and medium-sized enterprises [43]. From the perspective of environmental pressure, the larger the enterprise’s size, the more public attention it receives and the more pressure it has to bear in terms of environmental legality [28]. A number of studies have also found that an enterprise’s size has a significant impact on green innovation activities [42,43,44]. Thus, when environmental regulation triggers green innovation activities, large enterprises have a greater ability and willingness to undertake green innovation.

According to the life cycle theory, the life cycle of an enterprise basically follows the three stages of ‘growth–maturity–decline’ [45]. There can be significant differences in the impact of EMSC on green innovation for enterprises at different life stages. Specifically, enterprises in the growth stage need to seek a stable position in the industry due to their weak competitive ability. Green technology innovation may be the key to market breakthroughs [46]. Furthermore, firms in the growth phase are sensitive to policy changes due to their rapid development and can quickly adjust to green innovation when environmental regulations emerge. Compared with the growth stage, enterprises in the maturity stage have significantly improved their profitability and financing capacity [47]. However, due to the strong asset specificity developed during the development process, enterprises are not able to make timely adjustments when relevant environmental regulatory standards emerge [48]. Therefore, there is no significant impact of environmental regulations on the green innovation capability of enterprises in the maturity stage. For enterprises in the decline stage, issues such as declining sales, market share and profits, and institutional rigidity can lead to a decline in green innovation capacity, and even in the presence of environmental regulation, deteriorating financial conditions can prevent enterprises from engaging in green innovation activities.

Based on the above analysis, following hypotheses were formulated for this study.

**H2:**  *EMSC can promote green innovation in SOEs.*

**H3:**  *EMSC can facilitate green innovation in large enterprises*.

**H4:**  *EMSC can promote green innovation in growth stage enterprises*.

#### 2.2.3. The Moderating Role of Stakeholders

According to the stakeholder theory, companies need to ensure their survival and growth by satisfying the demands of their stakeholders. Flore et al. (2014) classify stakeholders into self-focused and reciprocal types [49]. According to Zhang et al. (2020), meeting the environmental needs of stakeholders is one of the purposes of an enterprise’s green innovation [50]. Therefore, this study investigates the moderating role of stakeholders in the influence of environmental management system certification on corporate green innovation from the perspective of self-focused and reciprocal stakeholders.

Self-focused stakeholders, such as suppliers and customers, focus on the realization of their own benefits [51]. These stakeholders are the direct counterparts to the enterprise and usually have different knowledge or professional backgrounds and are willing to share information with the enterprise to enrich the knowledge base and promote the generation of new knowledge. Suppliers are more motivated to greening their operations if they receive a timely financial return, but they also need to invest more effort and cost, thus crowding out their own R&D investment in green innovation [52]. Maintaining good customer relationships not only helps customers to participate in EMSC and promote green innovation, but also helps customers to exert pressure on enterprises through their own purchasing behavior. Thus, among the self-focused stakeholders, the moderating role of suppliers in EMSC on corporate green innovation is not yet clear, while customer satisfaction positively moderates this process.

Reciprocal stakeholders are not directly involved in business transactions but can influence enterprise performance and decisions, such as shareholders, creditors, government, etc. [51]. Green innovation activities require enterprise changes in the organization of the supply chain and increased integration and sharing of information, all of which require the support of shareholders, creditors, and others. Under the pressure of environmental protection, if reciprocal stakeholders value green practices, the enterprise will receive stronger financial support by meeting its interests. Therefore, reciprocal stakeholders, such as shareholders and creditors, play a positive role in regulating the influence of EMSC on enterprises’ green innovation. In summary, this paper proposes the following research hypothesis.

**H5:**  *Customers, shareholders, and creditors will play a positive moderating role in the process of EMSC influencing corporate green innovation, while the moderating role played by suppliers needs to be further explored.*

## 3. Materials and Methods

### 3.1. Sample Selection and Data Sources

According to the classification of heavily polluting industries in the Management List of Environmental Protection Verification Industries for Listed Companies issued by the Ministry of Environmental Protection of China in 2008 and the Guideline on Industry Classification of Listed Companies issued by the China Securities Regulatory Commission in 2012, enterprises in 14 industries such as extractive industries and food and beverage industries were classified as heavy polluting enterprises. This study uses the data of listed companies of heavy polluters in Shanghai and Shenzhen A-shares from 2011–2018 as a sample. The data of EMSC in the sample enterprises were manually collected from the National Certification and Accreditation Information Public Service Platform, the green patent data of the enterprises were obtained from the CNRDS database, and other data were obtained from the CSMAR database. In the process of data processing, if a company’s name contains the ST, *ST, and SST symbols, the company is not doing well, so we exclude it from the sample, moreover, companies with data outliers and samples with missing data for some indicators are also excluded. In addition, to ensure the comparability of the data, this study also excluded companies whose year of EMSC was before 2011. A total of 555 companies with 3960 sample data were finally selected.

### 3.2. Variables Definition

#### 3.2.1. Explained Variable

This paper adopts a number of green invention patent applications to measure the green innovation capability of enterprises. The number of green invention patent applications is collected from the CNRDS database, green innovation metric of the enterprise was obtained after adding 1 to this value and taking the natural logarithm. The reason for choosing the number of green patent applications as the indicator of green innovation is that the application of green patents with a high technical threshold, which requires enterprises to develop, promote, and apply the corresponding green technology on the basis of improving the performance of their own products, thus, this value can better reflect the high-level green innovation capability [53].

#### 3.2.2. Explanatory Variable

The core explanatory variable in this paper is environmental management system certification (EMSC), which is equal to 1 if the firm is certified and 0 otherwise. Since initial certification incurs a large investment, enterprises that have been certified for environmental management systems tend to be certified again to retain their qualification, so for enterprise i, if it is certified for environmental management systems in year t, then from year t onwards and subsequent years, this variable takes the value of 1, otherwise, it takes the value of 0.

#### 3.2.3. Moderating Variables

(1) Supplier satisfaction. According to existing studies, the accounts payable turnover rate is used as a measure of supplier satisfaction [54], and the higher the turnover rate, that the shorter the time the company takes up the supplier’s payment, the less it takes up its funds, the more convenience it provides to the supplier, and the higher the degree of supplier satisfaction. (2) Customer satisfaction. Referring to Cheng et al. (2016), inventory turnover rate is used as a measure of customer satisfaction [38], and a higher turnover rate means that companies have more frequent dealings with customers, closer relationships, smoother cooperation, and a higher degree of customer satisfaction. (3) Shareholder satisfaction. Referring to Bernadette et al. (2001), measurement of shareholder satisfaction using earnings per share [55], the higher the value of this indicator, the higher the degree of shareholder satisfaction. (4) Creditor satisfaction. Creditor satisfaction is measured using a negative balance sheet ratio with reference to existing study [56].

#### 3.2.4. Control Variables

To reduce the omitted variable bias associated with the econometric model setting, this study adds a series of control variables to the model. These variables include 13 control variables such as enterprise size, ownership, age, cash holdings, cash flow level, return on assets, operating income growth rate, profit growth rate, capital intensity, market power, director size, executive compensation, and book-to-market ratio. The meanings and symbols of each variable are shown in Table 2.

### 3.3. Model Specification

Since different enterprises have been certified at different times, in order to better identify the relationship between EMSC and corporate green innovation, the following DID model was constructed with reference to Beck et al. (2010) [63] to incorporate the differences arising from different certification times into the study, and the model was constructed as follows.

(1) $$GIf_{i,t}=\beta_{0}+\beta_{1}EMS_{i,t}+\beta_{2}Control_{i,t}+\lambda_{i}+v_{t}+\varepsilon_{i,t}$$

Model (1) is a baseline regression model, where $GIf_{i,t}$ represents the green innovation status of enterprise *i* in year *t*. $EMS_{i,t}$ represents the EMSC status of enterprise *i* in year *t*, which is measured by using dummy variables. $Control_{i,t}$ represents the control variables, $\lambda_{i}$ is the enterprise fixed effect, $v_{t}$ is the year fixed effect, $\beta_{1}$ is the main parameter of interest in this paper, which reflects the relationship between environmental management system certification and corporate green innovation.

The DID method is based on the premise that both the treatment and control groups in the sample have a common trend prior to the certification of the environmental management system, that is the trend in green innovation capability is the same for all enterprises before certification. If the common trend assumption does not hold, the results estimated by the DID method prove to be unreliable. To test this parallel trend, model (2) was constructed by referring to Beck et al. [63].

(2) $$GIf_{i,t}=\beta_{0}+\beta_{1}EMS_{i,t}^{-3}+\beta_{2}EMS_{i,t}^{-2}+\ldots +\beta_{6}EMS_{i,t}^{3}+\lambda_{i}+v_{t}+\varepsilon_{i,t}$$

where, $EMS_{i,t}^{-3}$ denotes the dummy variables for 3 years before passing the EMSC, $EMS_{i,t}^{3}$ denotes the dummy variables for 3 years after passing the EMSC, and the meanings of other variables follow in the same way.

In order to test whether the hypothesis H5 is true, in reference to the existing study [64], the following model is constructed for mechanism analysis in this study.

(3) $$GIf_{i,t}=\beta_{0}+\beta_{1}EMS_{i,t}+\beta_{2}L_{x}+\beta_{3}EMS_{i,t}*L_{x}+\beta_{4}Control_{i,t}+\lambda_{i}+v_{t}+\varepsilon_{i,t}$$

In model (3), $L_{x}$ represents stakeholder satisfaction, and the value of *x* ranges from 1 to 4. $L_{1}$ represents supplier satisfaction, $L_{2}$ represents customer satisfaction, $L_{3}$ represents shareholder satisfaction, and $L_{4}$ represents creditor satisfaction. The meanings of other symbols in model (3) are consistent with model (1).

## 4. Results and Discussion

### 4.1. Descriptive Statistics

Table 3 shows the descriptive statistics of variables. It can be seen that the means and standard deviations of the explained variable are 1.902 and 5.098, respectively, indicating that the overall green innovation level of enterprises in China’s heavy pollution industry is not high and there are large differences in green innovation capabilities among enterprises. In addition, the standard deviations of enterprise size (Sca), enterprise age (Age), and profit growth rate (Ear) are 3.557, 6.819, and 4.311, respectively, indicating that there are large differences in asset size, years of listing, and profit growth rate among enterprises in China’s heavy pollution industry.

### 4.2. Impact of ESMC on Enterprises’ Green Innovation

To test hypothesis H1, model (1) is estimated using the DID method, and the estimation results are presented in Table 4. Column (1) does not control for year fixed effects, individual fixed effects, and other control variables. The estimation results in this column indicate that EMSC has a significant positive promotion effect on enterprises’ green innovation, however, this result has a large omitted variable bias. Therefore, columns (2) and (3) control for year fixed effects and individual fixed effects in turn, and the estimated coefficients do not fluctuate significantly and remain significantly positive at the 1% level. Column (4) adds control variables to column (3), and the coefficient is 0.169, which is significantly positive at the 1% level. This indicates that EMSC has a significant positive effect on green innovation, which means that enterprises can significantly increase their green patent applications through EMSC. Hypothesis H1 is proved.

### 4.3. Robustness Test

To further illustrate the effectiveness of EMSC in promoting green innovation in enterprises of heavy pollution industries in China, this paper introduces a series of robustness tests.

#### 4.3.1. Common Trend Test

**Figure 2:**
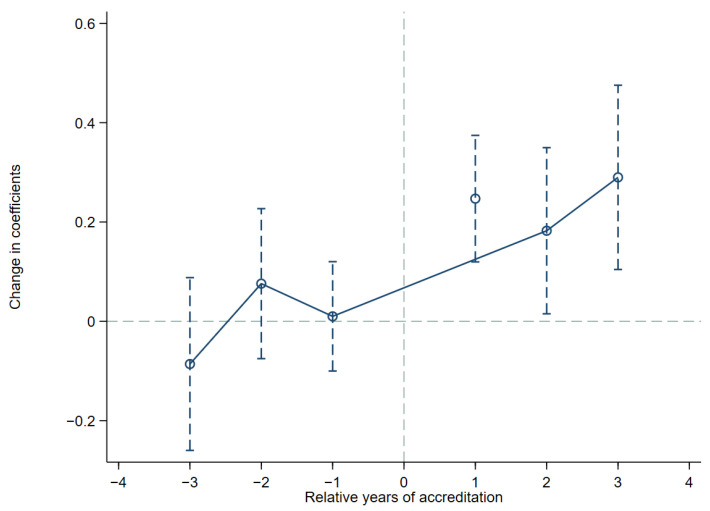
Common trend test.

The result of the common trend test using Model (2) is shown in Figure 2. For $GIf_{i,t}$, $\beta_{1}$–$\beta_{3}$ are not significantly different from zero, indicating that there is no significant difference among all enterprises before passing the EMSC, while after passing the EMSC, the model coefficients start to be significantly different from zero, indicating that there is a significant difference among enterprises after passing the EMSC. These results indicate that the model used in this paper passes the common trend test and shows that the estimation method of model (1) is feasible.

#### 4.3.2. Placebo Test

**Figure 3:**
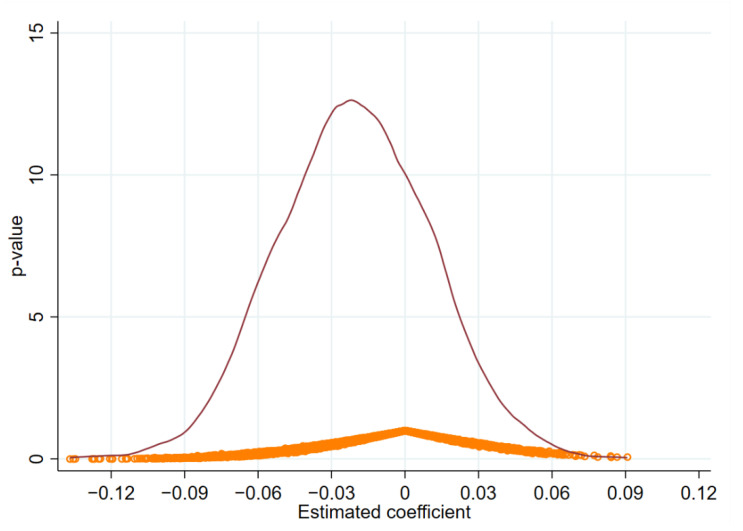
Placebo test.

The placebo test was introduced to discern the validity of the EMSC. Specifically, by retaining a sample of all enterprises for all years before the EMSC, 300 data were randomly selected in the year variable as a dummy year for the enterprises to pass the certification. To improve the identification ability, the experiment was repeated 6000 times, and the distribution of the estimated coefficients was obtained as shown in Figure 3. From Figure 3, it can be seen that all the estimated values approximately obey a normal distribution with zero mean and the *p*-value is less than 5%, which indicates that the effect of EMSC on enterprises’ green innovation is not found in the randomly selected “dummy certification passing years”, so the placebo test is passed.

#### 4.3.3. Substitution of Explanatory Variables

The total number of green patent applications (GIfA) was used to replace the number of green invention patent applications and brought into model (1) for re-estimation, and the results are shown in Table 5. From Table 5, we can see that the coefficient of the EMSC is significantly positive at the 1% level, which is consistent with the estimation results of Table 4. This shows that after replacing the explanatory variables, EMSC still has a significant promotion effect on enterprises’ green innovation.

#### 4.3.4. Change Observation Window Period

The three years before and after the official implementation of the New Environmental Protection Law were selected as the window period for testing, and the results are shown in Table 6. The effect of EMSC on enterprises’ green innovation is significantly positive at the 5% level, which is consistent with the previous findings. This means that after the adjustment of the observation window period, the EMSC still has a significant promotion effect on enterprises’ green innovation.

#### 4.3.5. Replacement Estimation Method

Considering that there are more cases where enterprises’ patents take zero value after taking a logarithm, this leads to the results obtained using the OLS method not being consistent estimators. Therefore, the Tobit model and the maximum likelihood estimation (MLE) method were used to re-estimate model (1), and the results are shown in Table 7. Columns (1) and (2) are the estimation results of the Tobit model and maximum likelihood estimation (MLE), respectively. The results in columns (1) and (2) indicate that EMSC promotes green innovation in enterprises, which is consistent with the estimation results in Table 4.

### 4.4. Heterogeneity Analysis

#### 4.4.1. Enterprises’ Sizes

The full sample was classified according to the “Statistical Classification of Large, Small, Medium and Micro Enterprises” issued by the National Bureau of Statistics in 2017, and the specific classification criteria and assigned values are shown in Table 8. Due to certain similarities in business philosophy and corporate strategies of small, medium, and micro enterprises, for statistical convenience, the three were combined for analysis.

After defining the enterprises’ sizes, the effects of EMSC on enterprises of different sizes were grouped and regressed according to model (1), and the estimation results are reported as shown in Table 9. The estimation results in column (1) of Table 9 show that the estimated coefficient of EMSC on green innovation capability of large enterprises is 0.170 and is significant at 1% significance level, implying that the green innovation level of large enterprises will increase by about 17% after the enterprises are certified by an environmental management system when other conditions remain unchanged; the estimation results in column (2) show that the estimated coefficient of EMSC on green innovation capability of small, medium, and micro enterprises (MSMEs) is 0.150 and significant at the 10% significance level, implying that the green innovation level of MSMEs will increase by about 15% after the certification of an environmental management system. Therefore, hypothesis H2 is proved.

#### 4.4.2. Enterprises’ Ownership

The sample was divided into two categories: state-owned enterprises, and non-state-owned enterprises, according to the enterprises’ ownership. The regression estimation results of the subgroups are shown in Table 10. From column (1), it can be seen that the estimated coefficient of EMSC on the green innovation capability of state-owned enterprises is 0.166 and is significant at the 5% significance level, indicating that the number of green innovation patent applications of state-owned enterprises will increase by about 16.6% after the enterprises have passed the EMSC when other conditions remain unchanged; from column (2), it can be seen that the estimated coefficient of EMSC on green innovation capability of non-state-owned enterprises is 0.145 and is significant at the 5% significance level, indicating that the number of green innovation patent applications of non-state-owned enterprises will increase by about 14.5% after the enterprises have passed the EMSC when other conditions remain unchanged. The estimation results in Table 10 indicate that the adoption of EMSC is beneficial for state-owned enterprises to carry out green innovation, and the positive promotion effect is slightly stronger than that of non-state-owned enterprises. Therefore, hypothesis H3 is proved.

#### 4.4.3. Enterprises’ Lifecycle

Referring to Gort et al. (1982) [65], this paper divides the enterprise life cycle into three periods of growth, maturity, and decline stage. The estimated results of the impact of green innovation capability on enterprises in different life cycles are reported in Table 11. The results in column (1) indicate that the estimated coefficient of EMSC for enterprises in the growth stage is 0.183 and is significant at the 1% significance level, implying that the green innovation capability of enterprises in the growth period is significantly promoted by EMSC when other conditions are constant; the results in columns (2) and (3) indicate that the estimated coefficients of EMSC for mature and declining stage enterprises are 0.081 and 0.237, respectively, indicating that the green innovation capability of mature and declining stage enterprises is also enhanced by EMSC, but it does not pass the significance test. This result indicates that EMSC can promote green innovation in mature-stage enterprises. Therefore, hypothesis H4 is proved.

### 4.5. Mechanism Analysis

The test results of hypothesis H5 are shown in Table 12, where columns (1) and (2) are the test of moderating effect of self-focused stakeholders and columns (3) and (4) are the test of moderating effect of reciprocal stakeholders. Columns (1) and (2) indicate the moderating effect of supplier satisfaction and customer satisfaction, respectively, and the results show that the interaction term of supplier satisfaction and EMSC has a suppressive effect on green innovation with a coefficient of −0.052 but does not pass the significance test, while the interaction term of customer satisfaction and EMSC has a positive effect on green innovation with a coefficient of 0.317 and is significant at 5%. Columns (3) and (4) indicate the moderating effects of shareholder satisfaction and creditor satisfaction, respectively, and the results show that the interaction terms of shareholder satisfaction, creditor satisfaction and EMSC all have a positive promotion effect on enterprises’ green innovation, with coefficients of 0.152 and 0.362, respectively, at the 5% significance level. Therefore, in the process of EMSC affecting enterprises’ green innovation, there are significant positive moderating effects of customer satisfaction, shareholder satisfaction, and creditor satisfaction, while the moderating effect of supplier satisfaction is not significant. Therefore, hypothesis H5 is proved.

### 4.6. Discussion

In recent years, the relationship between environmental regulation and green innovation has attracted more and more scholars’ attention. As a voluntary environmental regulation tool [28], however, little literature focuses on the relationship between EMSC and green innovation, many studies instead research the impact of EMSC on enterprises’ operations, function, performance, etc. [66,67,68,69]. In this paper, we further focus on the impact of EMSC on corporate green innovation based on the existing studies and find EMSC has a significant promotion effect on corporate green innovation, and this result is consistent with the weak Porter hypothesis [70], that is, environmental regulations lead to innovations. The result is also consistent with the study of Li et al. (2019) [71], that is, EMSC is significantly positively correlated with corporate green innovation. However, some differences exist. Firstly, we constructed a DID model to identify the causality between EMSC and green innovation instead of a simple multiple linear regression, which improves the confidence of the estimation results. Furthermore, on the mechanism analysis, we further analyzed the moderating role of stakeholders, this further clarifies the impact pathway of EMSC on enterprises’ green innovation.

## 5. Conclusions and Policy Implications

### 5.1. Conclusions

This paper constructs a DID model to explore whether EMSC can promote enterprises to carry out green innovation, taking listed companies of heavy polluters in A-shares from 2011–2018 as the research subject. The following findings are found:

(1) EMSC can significantly promote enterprises’ green innovation. The model in this paper has passed the placebo test and the common trend test, respectively, and the conclusions are still robust after changing the estimation method, explained variables, and observation window period. This shows that the participation of enterprises in environmental management system certification can help promote the development of green innovation and can better balance the relationship between economic benefits and ecological environmental protection.

(2) Compared with medium, small, and micro enterprises, EMSC of large-scale enterprises have a more significant promoting effect on green innovation. Large-scale enterprises have the basic conditions and internal environment to carry out green innovation, but medium, small, and micro enterprises are trapped by various restrictions and cannot adjust their innovation strategies in the short run. Compared with non-state-owned enterprises, state-owned enterprises have a stronger intention of green innovation through EMSC, as state-owned enterprises can often obtain strong support in terms of policies and resources. Enterprises in the growth stage can significantly promote green innovation with EMSC. Unlike from mature and declining companies, growth-stage companies urgently need to establish a stable position in the industry and green innovation is an important means to gain competitive advantage and achieve market breakthroughs. Besides this, growth-stage enterprises are more sensitive to policy changes and can quickly adjust their strategies under the constraints of environmental regulations.

(3) In the process of EMSC promoting green innovation of enterprises, customers, shareholders, and creditors can all have a significant positive moderating effect, while the moderating effect of suppliers has not passed the significance test. By satisfying the interests and demands of customers, shareholders, and creditors, enterprises can create a favorable environment for their own green innovation, thus, their green innovation level will be fostered. By satisfying the needs of suppliers, enterprises can help suppliers obtain economic returns in a timely manner and help them carry out green and environmentally friendly operations. However, enterprises also need to invest more resources in this, thus crowding out the R&D investment of green innovation projects. Therefore, the moderating effect of suppliers is not significant.

### 5.2. Policy Implications

Based on the conclusions of this paper, the following policy implications are obtained. (1) The enforcement of environmental regulations should be strengthened. The government should enhance the implementation of environmental regulations to reduce enterprises’ pollution emissions at the source. Market instruments should be used to stimulate enterprises to carry out green innovation and actively guide them to develop in the direction of innovation and environmental protection. The reform of state-owned enterprises to break the situation of lagging technological innovation due to industry monopoly should be promoted. The assessment mechanism of local governments should be optimized and the weight of public influence on performance assessment should be increased. (2) Improvement of the certification regulatory review system. Regulators should systematically assess the improvement of the environmental performance of enterprises before and after certification, and continuously pay attention to whether enterprises have the ability to meet standard environmental governance. In addition, regulators must adhere to an objective and impartial stance and not loosen their monitoring standards due to public opinion. New technologies should be actively introduced and a database of environmental information should be built. (3) Optimization of the corporate governance system. Enterprises need to develop reasonable management measures to protect their core competitiveness and build scientific and reasonable incentive mechanisms as well as supervision mechanisms, taking into account the life cycle stages, ownership, and size of enterprises. (4) Managers should pay attention to the interests of their stakeholders, especially customers, shareholders, and creditors, and these stakeholders have a significant impact on green innovation capabilities. Therefore, managers should regularly collect and summarize the appeals and policy requirements of these stakeholders, so as to provide both a basis for and help for enterprises to improve their green innovation capabilities.

However, limitations also exist. Firstly, in the empirical study, non-heavily polluting enterprises are not considered, thus, whether the results are effective to non-heavily polluting enterprises remains unknown. Secondly, we only conducted research on the impact of EMSC on enterprises’ green innovation, but there is another voluntary environmental regulation of Eco-Management and Audit System (EMAS) that should also be taken into account.

The following issues are worthy of further research: first, in terms of the research subjects, future research can include listed companies in non-heavy polluting industries to compare the differences between them and test whether the conclusions are universal. Second, this paper studies EMSC without considering the Eco-Management and Audit System (EMAS) certification issued by the EU, and there may be a substitution or crowding-out effect, resulting in an incomplete sample. Third, it can follow the idea of macro-level research by constructing a comprehensive evaluation index system for measurement.

## Figures and Tables

**Table 1:** Frequency of keywords and their year of first mention (Top 20).

No.	Key Words	Frequency	Year
1	performance	310	2008
2	innovation	282	2007
3	impact	275	2008
4	green innovation	230	2010
5	management	197	2008
6	corporate social responsibility	154	2008
7	environmental regulation	151	2009
8	green	148	2008
9	eco innovation	145	2014
10	strategy	118	2008
11	financial performance	116	2015
12	firm performance	112	2010
13	policy	108	2009
14	research and development	104	2008
15	sustainability	99	2014
16	empirical evidence	97	2016
17	determinant	91	2008
18	firm	90	2008
19	product innovation	86	2017
20	environmental performance	86	2011

**Table 2:** Symbol of each variable and their definition.

Variables	Symbol	Definition
Green Innovation	GIf	The number of green invention patents of enterprises plus one is taken as the natural logarithm [57]
Size	Sca	Natural logarithm of total assets [12]
Ownership	Own	1 for state-owned enterprises, 0 otherwise
Age	Age	Years on market/Total years
Cash hold	Cas	(Monetary funds + financial assets held for trading)/total assets [58]
Cash Flow Level	Flo	Net cash flow from operating activities/total assets [58]
Return on Assets	Pro	Net profit/Average total assets [59]
Business Growth	Inc	Operating income growth rate: (current period operating income–previous period operating income)/previous period operating income [58]
Profit growth rate	Ear	(Total profit for the current year − Total profit for the same period of the previous year)/(Total profit for the same period of the previous year)
Capital Intensity	Cap	ln(Total fixed assets/number of employees + 1) [60]
Market Power	Mar	ln(Operating income/operating cost) [59]
Board Size	Bor	Number of board members/number of independent directors
Executive Compensation	Com	Top three executives’ salaries are taken as logarithms
Book-to-market ratio	BM	Book value/Market value [61]
Supplier Satisfaction	L1	Accounts Payable Turnover Ratio [62]
Customer Satisfaction	L2	Inventory turnover rate [62]
Shareholder Satisfaction	L3	Earnings per share [62]
Creditor satisfaction	L4	Gearing ratio [62]

**Table 3:** Results of variable descriptive statistics.

Variable	N	Mean	Median	SD	Min	Max
GIf	3945	1.902	0.000	5.098	0.000	35.000
EMS	3945	0.152	0.000	0.359	0.000	1.000
Sca	3945	3.754	3.557	1.339	1.179	7.791
Own	3945	0.520	1.000	0.500	0.000	1.000
Age	3945	11.52	12.00	6.819	0.000	27.000
Cas	3945	0.159	0.123	0.124	0.010	0.608
Flo	3945	0.051	0.050	0.068	0.167	0.239
Pro	3945	0.038	0.034	0.063	0.216	0.213
Inc	3945	0.203	0.108	0.562	0.520	4.140
Ear	3945	−0.03	0.108	4.311	25.510	18.950
Cap	3945	1.835	1.680	0.884	0.269	4.758
Mar	3945	0.932	0.838	0.272	0.673	2.047
Bor	3945	8.859	9.000	1.792	5.000	15.000
Com	3945	2.655	2.639	0.679	0.942	4.510
BM	3945	0.628	0.636	0.256	0.099	1.142
L1	3945	0.198	0.049	1.997	0.000	91.360
L2	3945	0.136	0.069	0.355	0.000	13.510
L3	3945	0.345	0.250	0.636	5.019	8.599
L4	3945	0.446	0.441	0.299	0.007	10.080
L5	3945	0.007	0.004	0.017	0.002	0.489

**Table 4:** The impact of EMSC on green innovation.

	(1)	(2)	(3)	(4)
	lnGIf	lnGIf	lnGIf	lnGIf
EMS	0.211 ***	0.209 ***	0.172 ***	0.169 ***
	(0.054)	(0.053)	(0.055)	(0.055)
_cons	0.493 ***	0.469 ***	0.465 ***	0.613 ***
	(0.029)	(0.035)	(0.026)	(0.220)
Time fixed effects	NO	YES	YES	YES
Enterprise fixed effect	NO	NO	YES	YES
Control variables	NO	NO	NO	YES
N	3960	3960	3960	3945
R^2^	0.008	0.012	0.013	0.018

Note: *** denotes significant at 1% significance, and robust standard errors are in parentheses, as follows.

**Table 5:** Robustness test (1)—substitution of explanatory variables.

	(1)	(2)
	GIfA	GIfA
EMS	0.126 ***	0.130 ***
	(0.047)	(0.047)
_cons	0.736 ***	0.747 ***
	(0.031)	(0.209)
Time fixed effects	YES	YES
enterprises fixed effect	YES	YES
Control variables	NO	YES
N	3584	3572
R^2^	0.01	0.018

Note: *** denotes significant at 1% significance.

**Table 6:** Robustness test (2)—change window period.

	(3)	(4)
	GIf	GIf
EMS	0.098 **	0.097 **
	(0.047)	(0.047)
Time fixed effects	YES	YES
enterprises fixed effect	YES	YES
Control variables	NO	YES
_cons	0.494 ***	0.710 ***
	−0.024	−0.21
N	2688	2684
R^2^	0.007	0.013

Note: ***, ** denote significant at 1% and 5% significance, respectively.

**Table 7:** Robustness test (3)—replacement estimation method.

	(1)	(2)
	Tobit	MLE
EMS	0.200 ***	0.200 ***
	(0.058)	(0.055)
_cons	0.613 ***	0.613 ***
	(0.154)	(0.140)
Time fixed effects	YES	YES
Control variables	YES	YES
N	3945	3945

Note: *** denotes significant at 1% significance, the standard errors obtained from bootstrap sampling 300 times are shown in parentheses.

**Table 8:** Enterprise size classification criteria and assignment.

Enterprises’ Size	Classification Criteria	Assignment
Large enterprises	Engaged in ≥ 1000 people or business income ≥ 40,000 million yuan	1
Medium enterprises	300 people ≤ employees < 1000 and 20 million yuan ≤ operating income < 40,000 million yuan	0
Small enterprises	20 people ≤ employees < 300 people and 3 million yuan ≤ operating income < 20 million yuan	0
Micro enterprises	Employees < 20 and business revenue < 3 million	0

**Table 9:** The impact of environmental management system certification on green innovation in enterprises of different sizes.

	(1)	(2)
	Large Enterprises	MSMEs
EMS	0.170 ***	0.150 *
	(−0.059)	(−0.088)
_cons	0.649 **	0.988 *
	−0.269	−0.573
Time fixed effect	YES	YES
Enterprise fixed effects	YES	YES
Control variables	YES	YES
N	3628	317
R^2^	0.019	0.052

Note: ***, **, * denote significant at 1%, 5%, and 10% significance.

**Table 10:** Heterogeneity analysis of enterprises’ ownership.

	(1)	(2)
	State-Owned Enterprises	Non-State-Owned Enterprises
EMS	0.166 **	0.145 **
	(−0.083)	(−0.071)
_cons	0.257	0.945 ***
	(−0.299)	(−0.242)
Time fixed effect	YES	YES
Enterprise fixed effects	YES	YES
Control variables	YES	YES
N	2050	1895
R^2^	0.029	0.031

Note: ***, ** denote significant at 1% and 5% significance, respectively.

**Table 11:** Heterogeneity analysis of enterprises’ lifecycle.

	(1)	(2)	(3)
	Growth Stage	Maturity Stage	Decline Stage
EMS	0.183 **	0.081	0.237
	(−0.073)	(−0.092)	(−0.185)
_cons	0.412	0.523	0.957 ***
	(−0.271)	(−0.527)	(−0.299)
Time fixed effect	YES	YES	YES
Enterprise fixed effect	YES	YES	YES
Control variables	YES	YES	YES
N	1809	1507	629
R^2^	0.024	0.031	0.048

Note: ***, ** denote significant at 1% and 5% significance, respectively.

**Table 12:** Results of the stakeholder moderation effect test.

	Self-Focused		Reciprocal	
	(1)	(2)	(3)	(4)
EMS	0.310 ***	0.318 ***	0.315 ***	0.316 ***
	(−0.036)	(−0.036)	(−0.036)	(−0.036)
L1	−0.258 ***			
	(−0.054)			
L1 * EMS	−0.052			
	(−0.21)			
L2		−0.054		
		(−0.072)		
L2 * EMS		0.317 **		
		(−0.207)		
L3			−0.038	
			(−0.039)	
L3 * EMS			0.152 **	
			(−0.069)	
L4				−0.243 ***
				(−0.08)
L4 * EMS				0.362 **
				(−0.162)
_cons	0.362 ***	0.385 ***	0.382 ***	0.497 ***
	(−0.101)	(−0.102)	(−0.102)	(−0.107)
Time fixed effects	YES	YES	YES	YES
Enterprise fixed effects	YES	YES	YES	YES
Control variables	YES	YES	YES	YES
N	3945	3945	3945	3945
R^2^	0.135	0.13	0.13	0.132

Note: ***, **, * denote significant at 1%, 5%, and 10% significance, respectively.

## Data Availability

The data presented in this research are available on the website of the CSMAR database, and Chinese Research Data Services Platform (CNRDS). The resources are as follows: https://cn.gtadata.com; https://www.cnrds.com/. All websites were accessed on 30 August 2022.
